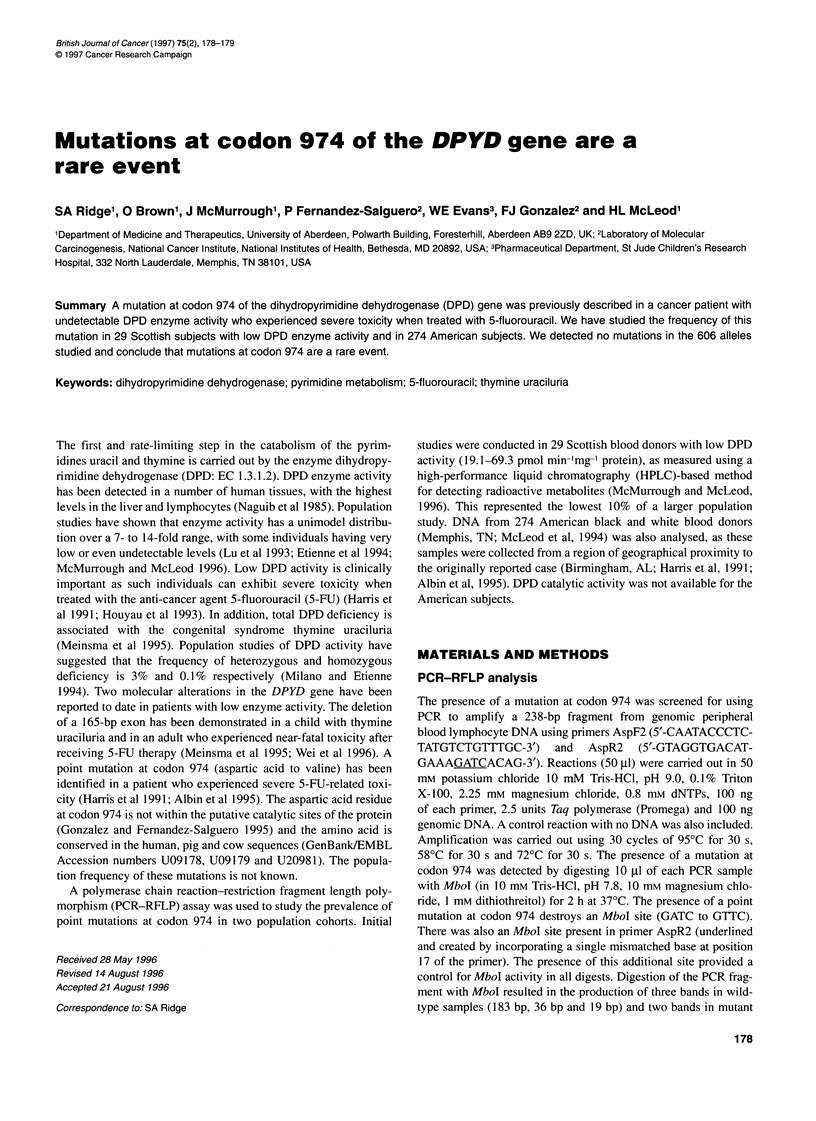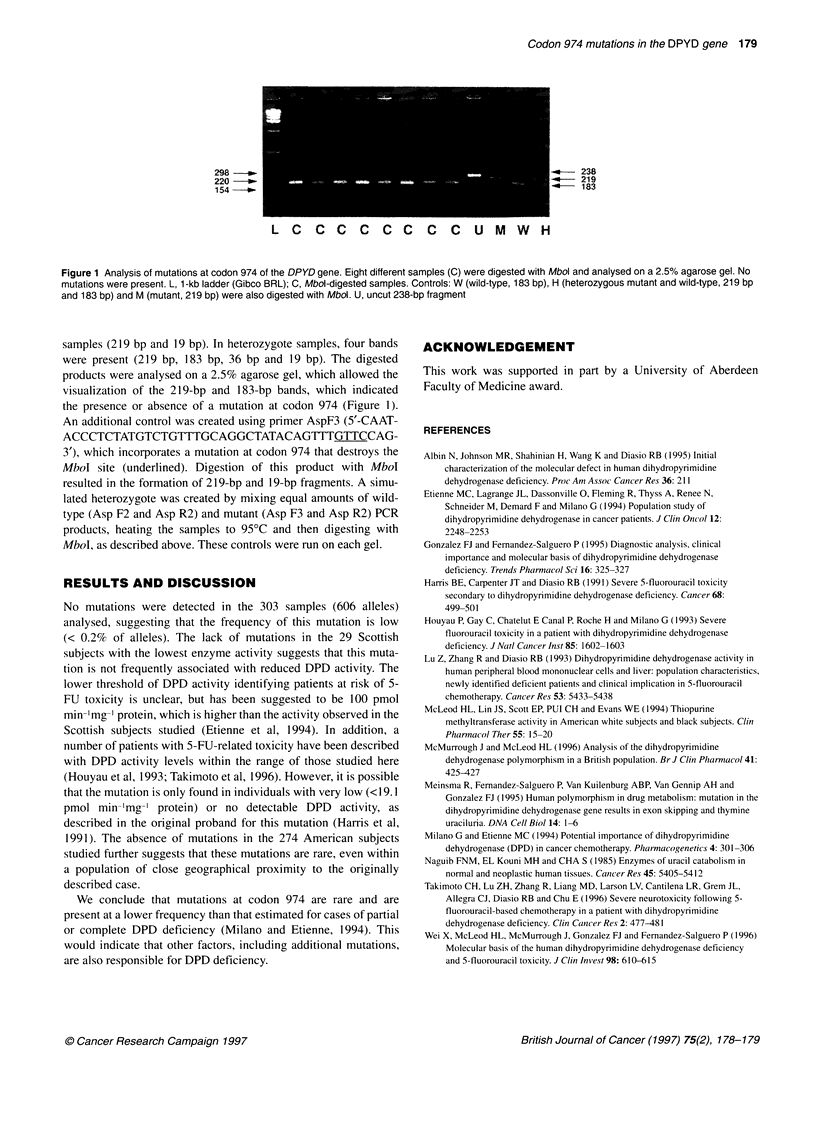# Mutations at codon 974 of the DPYD gene are a rare event.

**DOI:** 10.1038/bjc.1997.29

**Published:** 1997

**Authors:** S. A. Ridge, O. Brown, J. McMurrough, P. Fernandez-Salguero, W. E. Evans, F. J. Gonzalez, H. L. McLeod

**Affiliations:** Department of Medicine and Therapeutics, University of Aberdeen, Foresterhill, UK.

## Abstract

**Images:**


British Journal of Cancer (1997) 75(2), 178–179
© 1997 Cancer Research Campaign

### Mutations at codon 974 of the *DPYD* gene are a rare event

**SA Ridge[1], O Brown[1], J McMurrough[1], P Fernandez-Salguero[2], WE Evans[3], FJ Gonzalez[2] and HL McLeod[1]**

[1]Department of Medicine and Therapeutics, University of Aberdeen, Polwarth Building, Foresterhill, Aberdeen AB9 2ZD, UK; [2]Laboratory of Molecular Carcinogenesis, National Cancer Institute, National Institutes of Health, Bethesda, MD 20892, USA; [3]Pharmaceutical Department, St Jude Children's Research Hospital, 332 North Lauderdale, Memphis, TN 38101, USA

**Summary** A mutation at codon 974 of the dihydropyrimidine dehydrogenase (DPD) gene was previously described in a cancer patient with undetectable DPD enzyme activity who experienced severe toxicity when treated with 5-fluorouracil. We have studied the frequency of this mutation in 29 Scottish subjects with low DPD enzyme activity and in 274 American subjects. We detected no mutations in the 606 alleles studied and conclude that mutations at codon 974 are a rare event.

**Keywords:** dihydropyrimidine dehydrogenase; pyrimidine metabolism; 5-fluorouracil; thymine uraciluria

The first and rate-limiting step in the catabolism of the pyrimidines uracil and thymine is carried out by the enzyme dihydropyrimidine dehydrogenase (DPD: EC 1.3.1.2). DPD enzyme activity has been detected in a number of human tissues, with the highest levels in the liver and lymphocytes (Naguib et al 1985). Population studies have shown that enzyme activity has a unimodel distribution over a 7- to 14-fold range, with some individuals having very low or even undetectable levels (Lu et al 1993; Etienne et al 1994; McMurrough and McLeod 1996). Low DPD activity is clinically important as such individuals can exhibit severe toxicity when treated with the anti-cancer agent 5-fluorouracil (5-FU) (Harris et al 1991; Houyau et al 1993). In addition, total DPD deficiency is associated with the congenital syndrome thymine uraciluria (Meinsma et al 1995). Population studies of DPD activity have suggested that the frequency of heterozygous and homozygous deficiency is 3% and 0.1% respectively (Milano and Etienne 1994). Two molecular alterations in the *DPYD* gene have been reported to date in patients with low enzyme activity. The deletion of a 165-bp exon has been demonstrated in a child with thymine uraciluria and in an adult who experienced near-fatal toxicity after receiving 5-FU therapy (Meinsma et al 1995; Wei et al 1996). A point mutation at codon 974 (aspartic acid to valine) has been identified in a patient who experienced severe 5-FU-related toxicity (Harris et al 1991; Albin et al 1995). The aspartic acid residue at codon 974 is not within the putative catalytic sites of the protein (Gonzalez and Fernandez-Salguero 1995) and the amino acid is conserved in the human, pig and cow sequences (GenBank/EMBL Accession numbers U09178, U09179 and U20981). The population frequency of these mutations is not known.

A polymerase chain reaction–restriction fragment length polymorphism (PCR–RFLP) assay was used to study the prevalence of point mutations at codon 974 in two population cohorts. Initial studies were conducted in 29 Scottish blood donors with low DPD activity (19.1–69.3 pmol $min^{-1}mg^{-1}$ protein), as measured using a high-performance liquid chromatography (HPLC)-based method for detecting radioactive metabolites (McMurrough and McLeod, 1996). This represented the lowest 10% of a larger population study. DNA from 274 American black and white blood donors (Memphis, TN; McLeod et al, 1994) was also analysed, as these samples were collected from a region of geographical proximity to the originally reported case (Birmingham, AL; Harris et al, 1991; Albin et al, 1995). DPD catalytic activity was not available for the American subjects.

*Received 28 May 1996*
*Revised 14 August 1996*
*Accepted 21 August 1996*

*Correspondence to:* SA Ridge

### MATERIALS AND METHODS

### PCR–RFLP analysis

The presence of a mutation at codon 974 was screened for using PCR to amplify a 238-bp fragment from genomic peripheral blood lymphocyte DNA using primers AspF2 (5′-CAATACCCTC-TATGTCTGTTTGC-3′) and AspR2 (5′-GTAGGTGACAT-GAAAGATCACAG-3′). Reactions (50 µl) were carried out in 50 mM potassium chloride 10 mM Tris-HCl, pH 9.0, 0.1% Triton X-100, 2.25 mM magnesium chloride, 0.8 mM dNTPs, 100 ng of each primer, 2.5 units *Taq* polymerase (Promega) and 100 ng genomic DNA. A control reaction with no DNA was also included. Amplification was carried out using 30 cycles of 95°C for 30 s, 58°C for 30 s and 72°C for 30 s. The presence of a mutation at codon 974 was detected by digesting 10 µl of each PCR sample with *Mbo*I (in 10 mM Tris-HCl, pH 7.8, 10 mM magnesium chloride, 1 mM dithiothreitol) for 2 h at 37°C. The presence of a point mutation at codon 974 destroys an *Mbo*I site (GATC to GTTC). There was also an *Mbo*I site present in primer AspR2 (underlined and created by incorporating a single mismatched base at position 17 of the primer). The presence of this additional site provided a control for *Mbo*I activity in all digests. Digestion of the PCR fragment with *Mbo*I resulted in the production of three bands in wild-type samples (183 bp, 36 bp and 19 bp) and two bands in mutant

**Figure 1** Analysis of mutations at codon 974 of the *DPYD* gene. Eight different samples (C) were digested with *Mbo*I and analysed on a 2.5% agarose gel. No mutations were present. L, 1-kb ladder (Gibco BRL); C, *Mbo*I-digested samples. Controls: W (wild-type, 183 bp), H (heterozygous mutant and wild-type, 219 bp and 183 bp) and M (mutant, 219 bp) were also digested with *Mbo*I. U, uncut 238-bp fragment samples (219 bp and 19 bp). In heterozygote samples, four bands were present (219 bp, 183 bp, 36 bp and 19 bp). The digested products were analysed on a 2.5% agarose gel, which allowed the visualization of the 219-bp and 183-bp bands, which indicated the presence or absence of a mutation at codon 974 (Figure 1). An additional control was created using primer AspF3 (5′-CAAT-ACCCTCTATGTCTGTTTGCAGGCTATACAGTTTGTTCCAG-3′), which incorporates a mutation at codon 974 that destroys the *Mbo*I site (underlined). Digestion of this product with *Mbo*I resulted in the formation of 219-bp and 19-bp fragments. A simulated heterozygote was created by mixing equal amounts of wild-type (Asp F2 and Asp R2) and mutant (Asp F3 and Asp R2) PCR products, heating the samples to 95°C and then digesting with *Mbo*I, as described above. These controls were run on each gel.

### RESULTS AND DISCUSSION

No mutations were detected in the 303 samples (606 alleles) analysed, suggesting that the frequency of this mutation is low (< 0.2% of alleles). The lack of mutations in the 29 Scottish subjects with the lowest enzyme activity suggests that this mutation is not frequently associated with reduced DPD activity. The lower threshold of DPD activity identifying patients at risk of 5-FU toxicity is unclear, but has been suggested to be 100 pmol $min^{-1}mg^{-1}$ protein, which is higher than the activity observed in the Scottish subjects studied (Etienne et al, 1994). In addition, a number of patients with 5-FU-related toxicity have been described with DPD activity levels within the range of those studied here (Houyau et al, 1993; Takimoto et al, 1996). However, it is possible that the mutation is only found in individuals with very low (<19.1 pmol $min^{-1}mg^{-1}$ protein) or no detectable DPD activity, as described in the original proband for this mutation (Harris et al, 1991). The absence of mutations in the 274 American subjects studied further suggests that these mutations are rare, even within a population of close geographical proximity to the originally described case.

We conclude that mutations at codon 974 are rare and are present at a lower frequency than that estimated for cases of partial or complete DPD deficiency (Milano and Etienne, 1994). This would indicate that other factors, including additional mutations, are also responsible for DPD deficiency.

### ACKNOWLEDGEMENT

This work was supported in part by a University of Aberdeen Faculty of Medicine award.

### REFERENCES

Albin N, Johnson MR, Shahinian H, Wang K and Diasio RB (1995) Initial characterization of the molecular defect in human dihydropyrimidine dehydrogenase deficiency. *Proc Am Assoc Cancer Res* **36**: 211

Etienne MC, Lagrange JL, Dassonville O, Fleming R, Thyss A, Renee N, Schneider M, Demard F and Milano G (1994) Population study of dihydropyrimidine dehydrogenase in cancer patients. *J Clin Oncol* **12**: 2248–2253

Gonzalez FJ and Fernandez-Salguero P (1995) Diagnostic analysis, clinical importance and molecular basis of dihydropyrimidine dehydrogenase deficiency. *Trends Pharmacol Sci* **16**: 325–327

Harris BE, Carpenter JT and Diasio RB (1991) Severe 5-fluorouracil toxicity secondary to dihydropyrimidine dehydrogenase deficiency. *Cancer* **68**: 499–501

Houyau P, Gay C, Chatelut E Canal P, Roche H and Milano G (1993) Severe fluorouracil toxicity in a patient with dihydropyrimidine dehydrogenase deficiency. *J Natl Cancer Inst* **85**: 1602–1603

Lu Z, Zhang R and Diasio RB (1993) Dihydropyrimidine dehydrogenase activity in human peripheral blood mononuclear cells and liver: population characteristics, newly identified deficient patients and clinical implication in 5-fluorouracil chemotherapy. *Cancer Res* **53**: 5433–5438

McLeod HL, Lin JS, Scott EP, PUI CH and Evans WE (1994) Thiopurine methyltransferase activity in American white subjects and black subjects. *Clin Pharmacol Ther* **55**: 15–20

McMurrough J and McLeod HL (1996) Analysis of the dihydropyrimidine dehydrogenase polymorphism in a British population. *Br J Clin Pharmacol* **41**: 425–427

Meinsma R, Fernandez-Salguero P, Van Kuilenburg ABP, Van Gennip AH and Gonzalez FJ (1995) Human polymorphism in drug metabolism: mutation in the dihydropyrimidine dehydrogenase gene results in exon skipping and thymine uraciluria. *DNA Cell Biol* **14**: 1–6

Milano G and Etienne MC (1994) Potential importance of dihydropyrimidine dehydrogenase (DPD) in cancer chemotherapy. *Pharmacogenetics* **4**: 301–306

Naguib FNM, EL Kouni MH and CHA S (1985) Enzymes of uracil catabolism in normal and neoplastic human tissues. *Cancer Res* **45**: 5405–5412

Takimoto CH, Lu ZH, Zhang R, Liang MD, Larson LV, Cantilena LR, Grem JL, Allegra CJ, Diasio RB and Chu E (1996) Severe neurotoxicity following 5-fluorouracil-based chemotherapy in a patient with dihydropyrimidine dehydrogenase deficiency. *Clin Cancer Res* **2**: 477–481

Wei X, McLeod HL, McMurrough J, Gonzalez FJ and Fernandez-Salguero P (1996) Molecular basis of the human dihydropyrimidine dehydrogenase deficiency and 5-fluorouracil toxicity. *J Clin Invest* **98:** 610–615

© Cancer Research Campaign 1997